# Validation of a new prognostic index score for disseminated nasopharyngeal carcinoma

**DOI:** 10.1038/sj.bjc.6602525

**Published:** 2005-04-05

**Authors:** C-K Toh, D Heng, Y-K Ong, S-S Leong, J Wee, E-H Tan

**Affiliations:** 1 Department of Medical Oncology, National Cancer Centre, 11 Hospital Drive, Singapore 169610, Singapore; 2 Clinical Trials and Epidemiology Research Unit, Singapore Health Services Pte Ltd, 11 Third Hospital Avenue, Singapore 168751, Singapore; 3 Department of Therapeutic Radiology, National Cancer Centre, 11 Hospital Drive, Singapore 169610, Singapore

**Keywords:** nasopharyngeal carcinoma, prognostic factors, metastatic survival

## Abstract

Patients with metastatic nasopharyngeal carcinoma have variable survival outcomes. We previously designed a scoring system to better prognosticate these patients. Here, we report results on validation of this new prognostic index score in a separate cohort of patients. Clinical features and laboratory parameters were examined in 172 patients with univariate and multivariate analyses and a numerical score was derived for each independent prognostic variable. Significant independent prognostic variables and their scores assigned included poor performance status (score 5), haemoglobin <12 g dl^−1^ (score 4) and disease-free interval (DFI) (DFI⩽6 months (score 10) or metastases at initial diagnosis (score 1)). Maximum score was 19 and patients stratified into three prognostic groups: good, 0–3; intermediate, 4–8; poor, ⩾9. When applied to a separate cohort of 120 patients, 59 patients were good, 43 intermediate and 18 poor prognosis, with median survivals of 19.6 (95% CI 16.1, 23.1), 14.3 (95% CI 12.3, 16.2) and 7.9 (95% CI 6.6, 9.2) months, respectively. (logrank test: *P*=0.003). We have validated a new prognostic score with factors readily available in the clinics. This simple score will prove useful as a method to prognosticate and stratify patients as well as to promote consistent reporting among clinical trials.

Nasopharyngeal carcinoma (NPC) occurs sporadically in the West but constitute a major health problem in several parts of Asia, including Southern China, Hong Kong and Singapore (Parkin, 2001). In Singapore, the incidence rate in Chinese is among the highest in Asia and the disease ranks as the fifth most common cancer among Chinese males (Chia *et al*, 2000). The histological pattern of NPC among the Chinese population comprise mainly the World Health Organization (WHO) types II (nonkeratinising) and III (undifferentiated) (Chan *et al*, 1998). Both histological types are often considered together as they share similar epidemiological, clinical and serologic characteristics, as distinct from the epidermoid carcinoma of the head and neck region seen in the western population (Shanmugaratnam and Sobin, 1993; Altun *et al*, 1995).

There is a high incidence of distant metastases with nonkeratinising or undifferentiated NPC, as compared to other epidermoid carcinoma arising in the head and neck region (Reddy *et al*, 1995). This is especially true for patients who present with locally advanced disease (Ahmad and Stefani, 1986). A prospective study showed a high rate of subclinical distant metastasis, with a distinctive feature of bone marrow invasion (Micheau *et al*, 1987). In addition, the incidence of distant failure after radiotherapy treatment for locally advanced disease can be as high as 57% in N_3_ disease (Lee *et al*, 1992). In view of the high rate of systemic relapse, chemotherapy has been incorporated into the primary treatment of locally advanced disease in order to improve the outcome. Concurrent chemoradiotherapy was shown to improve overall and progression-free survival for locally advanced NPC in the Intergroup study (Al-Sarraf *et al*, 1998) and this was replicated in studies carried out in the endemic areas of Singapore (Wee *et al*, 2004), Hong Kong (Lee *et al*, 2004) and Taiwan (Lin *et al*, 2003). However, a significant proportion of patients would still relapse systemically despite combined modality therapy and many of these patients would ultimately succumb to disseminated disease.

Patients with disseminated disease do not behave in a uniform manner. It is hence not surprising to see significantly variable results between studies of similar therapeutic manoeuvres in patients with metastatic NPC. We have shown in our previous study that by using several clinical and laboratory parameters, we were able to define three prognostically distinct groups of patients with disseminated NPC (Ong *et al*, 2003). We proposed that a prognostic index scoring system using these parameters could be used for a more accurate prognostic evaluation of a patient. However more importantly, it can also be used for a more accurate stratification of patients in prospective clinical studies and hopefully help to standardise reporting results of any therapeutic interventions.

We now report a follow-on study that reanalysed our previous findings to define a new prognostic index score and to validate this new score in a separate cohort of patients with disseminated NPC.

## PATIENTS AND METHODS

### Patients

All patients were treated at the Department of Medical Oncology, National Cancer Centre between January 1994 and January 2003. There were two different cohorts of patients: the first (Cohort 1) was the group on which the new prognostic index score was derived and included 220 patients treated between January 1994 and December 1999, while the second cohort (Cohort 2) was the group on which the new score was validated. Cohort 2 included 99 patients treated between January 2002 and January 2003 and 21 patients (not included in our previous analysis for Cohort 1) from a previous Institutional Review Board-approved phase II clinical trial conducted in 1996 (Au *et al*, 1998).

All patients had a histological confirmation of NPC and had computerised tomography (CT) scan of the posterior nasal space, chest X-ray and/or CT scan of the thorax, ultrasound or CT scan of the abdomen and bone radionuclide scan to identify the extent of systemic disease. Patients were classified into the International Union Against Cancer/American Joint Committee on Cancer (UICC/AJCC) stages using the clinical and radiological data.

Pretreatment patient and disease characteristics, disease-free interval (DFI) (time from the onset of primary radiotherapy or chemoradiotherapy to the time of distant relapse), type of chemotherapy given, best response to chemotherapy, type of salvage chemotherapy given and date of death were recorded for all patients.

### Survival data

The primary end point of interest was metastatic survival. Metastatic survival was defined as survival subsequent to the development of distant relapse, that is, from the first diagnosis of distant metastases to the time of death. Locoregional recurrence was not considered a distant relapse. The survival status of all patients was verified with Singapore's national death registry for Cohorts 1 and 2 as on 30 June 2000 and 31 December 2003, respectively. The cohorts excluded patients who were nonresidents of Singapore and as notification is mandatory in the event of death, the mortality data obtained from the death registry was complete and exhaustive.

### Statistical analysis

The analysis to derive a new prognostic score was performed on Cohort 1 and focused only on 172 patients who had received chemotherapy. The original prognostic index score was based on all patients including patients who were not treated with chemotherapy (Ong *et al*, 2003). As chemotherapy has an impact on survival outcome in patients with disseminated disease, there was a need to rederive a new prognostic index score using only patients from Cohort 1 who received palliative chemotherapy as all the patients from Cohort 2 were treated with chemotherapy. Univariate and multivariable analyses were performed using the Cox proportion hazards model. The multivariable analyses were undertaken with both forward and backward stepwise procedures for identifying the independent prognostic variables. Factors that were considered in the derivation of the new prognostic index score included age, gender, performance status according to Eastern Co-operative Oncology Group (ECOG) criteria, specific metastatic sites, number of metastatic sites, metastasis at presentation, stage at first diagnosis, DFI, leucocyte count, haemoglobin (Hb) level, and albumin level, on the basis of our previous analysis. In order to construct the new prognostic index score, factors were entered as categorical values as far as possible to keep the computations simple, although categorisation inevitably will result in some loss of information. *P*⩽0.05 was used as the cutoff value of statistical significance for variable selection in the multivariable modelling. The regression coefficient of each independent prognostic variable (the *β* in the Cox regression equation hazard ratio (HR)=e^*β*^) is then modified into an integer numerical value to construct the new prognostic index score. The patients were stratified, based on the new prognostic index score, into three different risk groups with significantly different median metastatic survivals.

The validation of the new prognostic index score was subsequently performed on Cohort 2 patients. Overall and median metastatic survival estimates and curves were obtained using the Kaplan–Meier method and logrank test was used to compare among the three prognostic groups stratified by the new score.

## RESULTS

### Patient and disease characteristics (Table 1)

Table 1 shows the characteristics of patients in both cohorts who had received at least one line of palliative chemotherapy at diagnosis of distant metastases. All patients had nonkeratinising or undifferentiated NPC. There was a male predominance in both cohorts. Most characteristics were similar between both cohorts. The median age at diagnosis of metastases was 47 years for Cohort 1 and 48 years for Cohort 2. In all, 35 (20.3%) patients in Cohort 1 and 27 (22.5%) patients in Cohort 2 had metastases at diagnosis. The majority had distant relapse after treatment for locally advanced disease previously. Bone was the most common site of metastasis, followed by liver, lung and distal nodes. Most of the patients had multiple sites involved at diagnosis of metastases.

The proportion of patients with good ECOG status of 0 and 1 was higher in Cohort 2 (95.8%) compared to Cohort 1 (87.8%) (*P*=0.07). Several chemotherapy regimens were used in the first-line setting and these included Cisplatin and Fluorouracil, Paclitaxel and Gemcitabine either alone or in combination with Carboplatin.

### Survival distribution

**Figure 1:**
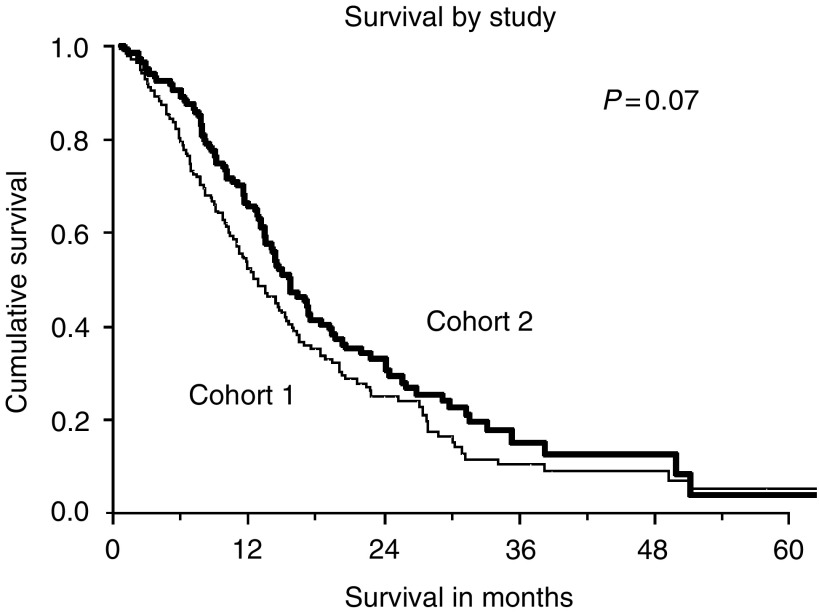
Survival curves for Cohorts 1 and 2. There is no statistically significant difference in metastatic survival between the two cohorts.

Patients from Cohort 2 appeared to have better survival, although this was not statistically significant (logrank test *P*-value=0.07). See Figure 1. The median metastatic survival for patients in Cohort 1 was 12.9 months (95% CI 10.5,15.3) and that for patients in Cohort 2 was 15.6 months (95% CI 13.2, 18.0). The 1-, 2- and 3-year survival proportions were 52, 25 and 10% for Cohort 1 and 66, 33 and 15% for Cohort 2, respectively.

### Univariate and multivariate analysis

The univariate analysis was performed on the 172 patients who were treated with chemotherapy in Cohort 1. Factors considered in the univariate analyses were based on our previous study and included age, gender, performance status, laboratory parameters such as Hb, leucocyte count and albumin level, stage at first diagnosis, sites and number of metastases and DFI. The factors associated with an adverse prognosis were anaemia (Hb<12g dl^−1^), ECOG ⩾2, DFI⩽6 months, presence of multiple metastases and liver metastases. See Table 2.

The variables included in the multivariate analysis were similar to that used in the univariate analysis. Using a significance level of 0.05, the significant independent variables were Hb level (Hb<12g dl^−1^ with HR: 2.1), performance status (ECOG⩾2 with HR: 2.6), DFI (metastasis at diagnosis and short DFI with HR: 1.2 and 7.7 respectively). See Table 3. These three variables predicted negatively for metastatic survival.

### New prognostic index score and risk groups

**Figure 2:**
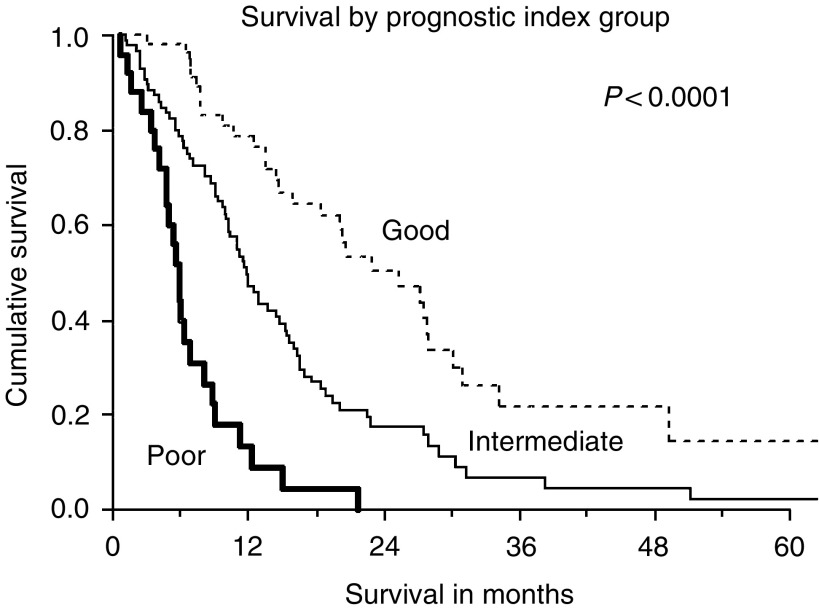
Survival by new prognostic index grouping derived from patients in Cohort 1. There is a clear demarcation of survival differences between the three risk groups.

A numerical score was derived from the regression coefficient of each of the three independent prognostic variables derived above, namely, anaemia (Hb<12 g dl^−1^), ECOG⩾2 and DFI. A score of 0 was assigned if the factor was absent or 1, 4, 5 or 10 according to the factor present. See Table 4. The new prognostic index score for each individual patient was calculated by adding up the scores of each independent factor. The maximum score obtainable was 19, instead of 20 as metastasis at diagnosis and short DFI were mutually exclusive. In view of the gaps between the scores, the possible scores were 0–1, 4–6, 9–11, 14–16 and 19. The patients were stratified into three prognostic groups based on the new prognostic index score: 57 patients in the low-risk group (score: 0–3), 86 patients in intermediate-risk group (score: 4–8) and 25 patients in high-risk group (score: ⩾9). The median metastatic survivals for the three different risk groups were 25.3 (95% CI 17.7–33.9), 11.7 (95% CI 9.9–13.6) and 5.8 (95% CI 5.0–6.5) months, respectively. (logrank test, *P*<0.0001). See Figure 2.

### Validation of new prognostic index score

**Figure 3:**
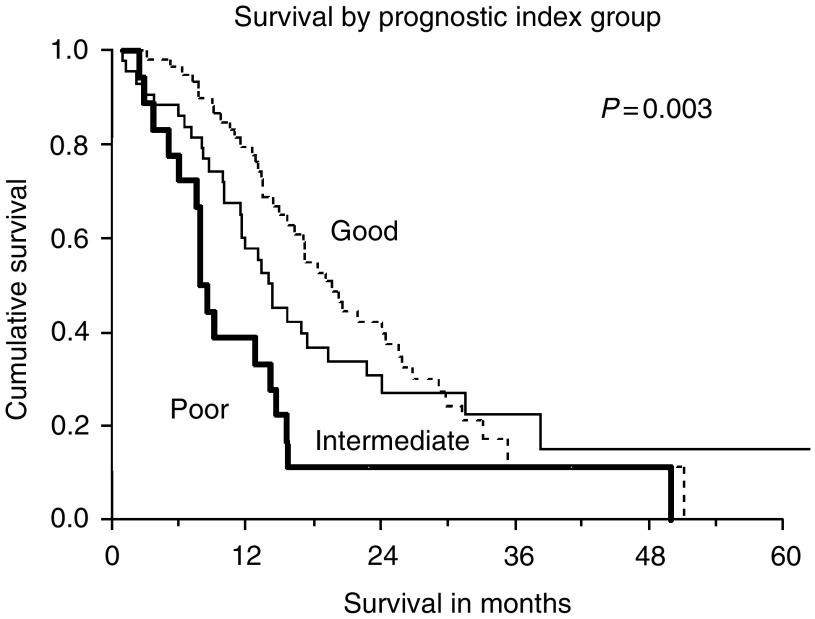
Survival by new prognostic index grouping applied to patients in Cohort 2. There is a statistically significant difference between the three risk groups.

The new prognostic index score was applied to the patients in Cohort 2. The proportion of patients in the low-risk, intermediate-risk and high-risk groups were 49, 36 and 15%, respectively. The median metastatic survivals of these three different groups were as follows: 19.6 (95% CI 16.1–23.1), 14.3 (95% CI 12.3–16.2) and 7.9 (95% CI 6.6–9.2) months respectively (logrank, *P*=0.003). See Figure 3.

## DISCUSSION

We previously presented our results on derivation of a prognostic index score derived from all patients in Cohort 1 that included those not treated with chemotherapy (Ong *et al*, 2003). However, as Cohort 2 was used for validation of the score and all the patients in Cohort 2 were treated, we elected to reanalyse Cohort 1 to restrict the derivation of a new prognostic index score to only patients given chemotherapy. The reason for the reanalysis is that chemotherapy has an impact on survival outcome in patients with disseminated NPC, although there are no randomised comparison studies between chemotherapy and best supportive care (Fandi *et al*, 1994; Hong *et al*, 1999). This is also supported by the fact that incorporation of chemotherapy in locally advanced disease has been shown to improve progression-free and overall survival (Al-Sarraf *et al*, 1998; Lin *et al*, 2003; Wee *et al*, 2004). Thus, with reanalysis, we have eliminated the bias with the use of chemotherapy and make the two cohorts more comparable.

We found that DFI, Hb level (<12g dl^−1^) and poor performance status (ECOG⩾2) were significant negative prognostic factors in patients treated with chemotherapy and these three independent variables were used in the derivation of the new prognostic index score. Metastasis at diagnosis and short DFI were regarded as two separate categories within the same DFI variable and thus were mutually exclusive. The natural history of a disease is dependent on the interaction of both patient and disease factors. Anaemia can be related to the disease process itself, host-related factors or treatment given and has been shown to be associated with significant reduction in survival in various cancers, other than NPC (Caro *et al*, 2001; Bokemeyer *et al*, 2002). Anaemia may reflect not only a biologically more aggressive tumour but may be a mediating factor to resistance to treatment and this has been demonstrated in retrospective studies on cervical cancer treated with chemoradiation (Obermair *et al*, 2001). The negative prognostic factor of a poor performance status has been shown in many other tumour types as well (Paesmans *et al*, 1995; Polee *et al*, 2003; Motzer *et al*, 2004). Patients with poor performance status do not tolerate treatment well and it may also be a reflection of the more advanced state of the cancer.

Patients who present with metastasis at diagnosis can be a result of delay in seeking medical attention or more likely, a reflection of the aggressive nature of the disease such that it is widespread by the time the patient becomes symptomatic. Having a short DFI is also reflective of an aggressive disease and has been convincingly shown to portend a poorer outcome in other tumours such as ovarian cancer (Markman *et al*, 1991). Our analysis showed that a short DFI after initial radiotherapy or chemoradiotherapy for the primary tumour had a worse prognostic score compared to metastasis at diagnosis and this is likely due to emerging chemoresistant clones within the tumour of those patients who relapse shortly after treatment.

A study by Teo *et al* (1996) showed that short DFI, the presence of liver metastasis and age at diagnosis were prognostic for metastatic survival in metastatic NPC. Short DFI is included as a factor in our new prognostic index score but the other two variables were found to be not statistically significant on multivariate analysis. This may be a result of our population of patients being limited to only those treated with chemotherapy and the treatment likely negated the prognostic significance of age and site of metastasis. Statistical issues, such as variations in modelling procedure in small to moderate size data sets between the different studies, could be an alternative explanation.

The metastatic survival of patients with disseminated NPC treated with chemotherapy is highly variable and our findings provide further supportive evidence for that. In Cohort 1, at the time of analysis in June 2000, the metastatic survival varied from 1 to 72 months. Similarly, for Cohort 2, the metastatic survival duration varied from 1 month to 103 months: 10 patients died within 3 months of diagnosis of metastatic disease despite chemotherapy, while 13 patients are still alive after 2 years. In fact, one patient survived 8 years 7 months while another three lived beyond 4 years after diagnosis of metastatic disease. A number of studies have reported similar findings, with several long-term survivors with the use of chemotherapy in metastatic NPC (Chan *et al*, 1997; Fandi *et al*, 2000). Based on the results of our studies, it is possible for single-arm studies or randomised studies that are inadequately powered or stratified to report spuriously improved survival outcome with a new therapeutic intervention because of biased patient selection alone. As a result of this heterogeneity, it is imperative that a method be developed to better prognosticate and stratify patients for more accurate assessment of efficacy of any new therapeutic interventions.

It is important to note that the new prognostic index score resulted in clear demarcation of three prognostically different groups with nonoverlapping confidence intervals of the median survival durations and this demarcation was replicated in the separate cohort of patients. Another important point to note is that the new prognostic index score incorporates clinical data that are used in routine patient care in most institutions.

There is accumulating evidence for the use of biomarkers in the detection and prognostication of NPC. The biomarkers include circulating markers in the blood, such as Epstein–Barr virus (EBV) DNA (Chien *et al*, 2001) and CYFRA 21-1 (Ma *et al*, 2004), as well as tumour immunohistochemical markers such as the expression of multidrug-resistance protein (Hsu *et al*, 2002), epidermal growth factor receptors (Chua *et al*, 2004) and signal transducers and activators of transcription factors (Hsiao *et al*, 2003). Among them, plasma EBV DNA has been the most validated and has been shown to be a predictor of poor survival after radiotherapy for local disease (Chan *et al*, 2002). However, these biomarkers are not readily available in most institutions and they are yet to be validated as prognostic factors in the metastatic setting.

It is conceivable that similar prognostic classifications can be designed and used for other disseminated solid tumours as well, for instance breast or colorectal cancers. Such a prognostic classification based on combination of patient and laboratory factors may be able to categorise patients with disseminated disease more accurately as shown in NPC in this study and hence will be useful for stratifying patients in randomised studies.

We have validated a new prognostic index score in metastatic NPC patients treated with chemotherapy. This new prognostic index score can stratify patients into three different prognostic groups with significantly different median metastatic survivals. This prognostic classification system will be useful for more accurate prognostication of patients with disseminated NPC. In addition, it can prove useful in the design of clinical trials for metastatic NPC as it can more accurately stratify patients into groups with fairly consistent outcome and thus make the results more comparable and interpretable.

## Figures and Tables

**Table 1:** Patient and disease characteristics

	**Cohort 1**		**Cohort 2**		
**Characteristics**	**No. of patients**	**%**	**No. of patients**	**%**	***P*-value**
No. of patients	172		120		
					
*Survival status*					See logrank test
Dead	130	75.6	91	75.8	
Alive	42	24.4	29	24.2	
					
*Age, in years*					<0.001
Median	47		48		
Interquartile range	(40,54)		(42,54)		
					
*Gender*					0.32
Male	144	83.7	95	79.2	
Female	28	16.3	25	20.8	
					
*ECOG status* a					0.07
0	18	10.5	19	15.8	
1	133	77.3	96	80	
2	14	8.1	2	1.7	
3	6	3.5	3	2.5	
4	1	0.6	0	0	
					
*Laboratory parameters*					
Albumin (g l^−1^)					0.38
<40	128	74.4	90	75	
⩾40	32	18.6	29	24.1	
Hemoglobin (g l^−1^)					0.003
<12	106	61.6	54	45	
⩾12	63	36.6	66	55	
					
UICC/AJCC stage at first diagnosis^b^					0.002
I	5	2.9	2	1.7	
IIA	1	0.6	4	3.3	
IIB	31	18	4	3.3	
III	39	22.7	39	32.5	
IVA	20	11.6	16	13.3	
IVB	27	15.7	16	13.3	
IVC	33	19.2	27	22.5	
					
*Disease-free interval*					0.16
Mets at diagnosis	35	20.3	27	22.5	
⩽6 months	11	6.4	15	12.5	
>6 month	125	72.7	78	65	
					
*Sites of metastasis*					
Bone	118	68.6	79	65.8	0.26
Liver	74	43	63	52.5	0.12
Lung	65	37.8	51	42.5	0.41
Distant nodes	63	36.6	39	32.5	0.43
					
*No. of metastatic sites*					0.3
Single	69	40.1	41	34.2	
Multiple	103	59.9	79	65.8	
					
*Prior chemotherapy before mets*					0.58
Yes	14	8.1	12	10	
No	158	91.9	108	90	
					
*Salvage chemotherapy*					0.02
Yes	91	52.9	68	61.8	
No	81	47.1	42	38.2	

a ECOG status refers to performance status as defined by the Eastern Cooperative Oncology Group.

b UICC/AJCC refers to International Union against Cancer/American Joint Committee on Cancer.

**Table 2:** Univariate analysis of patients treated with chemotherapy in Cohort 1 (*N*=172)

**Factor**	**No. of patients**	**No. alive**	**Hazard ratio (95% CI)**	***P*-value**
*Gender*				
Female	28	6	Baseline	
Male	144	36	0.66 (0.41–1.04)	0.075
				
*Age, in years*				
⩽54	78	23	Baseline	
46–65	84	16	1.31 (0.92–1.88)	0.14
>65	10	3	1.79 (0.81–3.96)	0.15
				
*Albumin, g l* ^−*1*^				
⩾04	32	10	Baseline	
<40	128	31	1.43 (0.89–1.88)	0.13
				
*Haemoglobin, g dl* ^−*1*^				
⩾12	63	28	Baseline	
<12	106	14	2.61 (1.76–3.87)	<0.001
				
*ECOG status* a				
0–1	151	41	Baseline	
2	14	1	2.24 (1.26–4.00)	0.006
3–4	7	0	4.19 (1.93–9.18)	<0.001
				
*Leucocyte count, × 10*^*9*^ *l*^−*1*^				
<4	26	4	Baseline	
4–11	112	35	0.77 (0.48–1.24)	0.29
>11	31	3	1.46 (0.83–2.56)	0.19
				
UICC/AJCC stage at first diagnosis^b^				
I–II	37	8	Baseline	
III–IVB	86	23	1.21 (0.78–1.89)	0.38
IVC	33	9	1.41 (0.81–2.45)	0.22
				
*Bone metastasis*				
No	42	10	Baseline	
Yes	118	27	1.24 (0.82–1.85)	0.3
				
*Liver metastasis*				
No	97	26	Baseline	
Yes	74	16	1.60 (1.13–2.28)	0.008
				
*Lung metastasis*				
No	106	27	Baseline	
Yes	65	15	1.19 (0.83–1.70)	0.35
				
*Distal node metastasis*				
No	107	19	Baseline	
Yes	63	22	0.81 (0.56–1.18)	0.27
				
*Number of metastatic sites*				
Single	69	17	Baseline	
Multiple	103	25	1.55 (1.08–2.21)	0.017
				
*Disease-free interval*				
>6 months	125	32	Baseline	
⩽6 months	11	1	4.03 (2.02–8.02)	<0.001
Metastases at diagnosis	35	9	1.25 (0.81–1.94)	0.31

a ECOG status refers to performance status as defined by the Eastern Cooperative Oncology Group.

b UICC/AJCC refers to International Union against Cancer/American Joint Committee on Cancer.

**Table 3:** Significant independent variables from multivariate analysis for patients treated with chemotherapy in Cohort 1 (*N*=172)

**Factor**	**Hazard ratio (95% CI)**	***P*-value**
Haemoglobin level (<12 g dl^−1^)	2.067 (1.34–3.17)	0.001
Performance status (ECOG ⩾2^a^)	2.585 (1.45–4.59)	0.001
Disease-free interval		
Metastasis at diagnosis	1.21 (0.75–1.95)	0.031
⩽6 months	7.656 (2.23–26.25)	

a ECOG refers to performance status as defined by the Eastern Cooperative Oncology Group.

**Table 4:** New prognostic index score and risk groups

**Factor**	**Score**	***β* (hazard ratio=e** ^***β***^ **)^a^**
Haemoglobin level (<12 g dl^−1^)	4	0.73
Performance status (ECOG ⩾2^b^)	5	0.95
Disease-free interval		
Metastasis at diagnosis	1	0.19
⩽6 months	10	2.04
Maximum score	19	

Low risk: score 0–3; intermediate risk: score 4–8; high risk: score ⩾9.

a *β*=Regression Coefficient.

b ECOG refers to performance status as defined by the Eastern Cooperative Oncology Group.
